# Towards personalized cardiometabolic risk prediction: A fusion of exposome and AI

**DOI:** 10.1016/j.heliyon.2024.e40859

**Published:** 2024-12-20

**Authors:** Zeinab Shahbazi, Slawomir Nowaczyk

**Affiliations:** Center for Applied Intelligent Systems Research, Halmstad University, Sweden

**Keywords:** Cardiovascular disease, Exposome, Clinical records, Artificial intelligence, Machine learning

## Abstract

The influence of the exposome on major health conditions like cardiovascular disease (CVD) is widely recognized. However, integrating diverse exposome factors into predictive models for personalized health assessments remains a challenge due to the complexity and variability of environmental exposures and lifestyle factors. A machine learning (ML) model designed for predicting CVD risk is introduced in this study, relying on easily accessible exposome factors. This approach is particularly novel as it prioritizes non-clinical, modifiable exposures, making it applicable for broad public health screening and personalized risk assessments. Assessments were conducted using both internal and external validation groups from a multi-center cohort, comprising 3,237 individuals diagnosed with CVD in South Korea within twelve years of their baseline visit, along with an equal number of participants without these conditions as a control group. Examination of 109 exposome variables from participants' baseline visits spanned physical measures, environmental factors, lifestyle choices, mental health events, and early-life factors. For risk prediction, the Random Forest classifier was employed, with performance compared to an integrative ML model using clinical and physical variables. Furthermore, data preprocessing involved normalization and handling of missing values to enhance model accuracy. The model's decision-making process were using an advanced explainability method. Results indicated comparable performance between the exposome-based ML model and the integrative model, achieving AUC of 0.82(+/-)0.01, 0.70(+/-)0.01, and 0.73(+/-)0.01. The study underscores the potential of leveraging exposome data for early intervention strategies. Additionally, exposome factors significant in identifying CVD risk were pinpointed, including daytime naps, completed full-time education, past tobacco smoking, frequency of tiredness/unenthusiasm, and current work status.

## Graphical abstract

**Figure:**
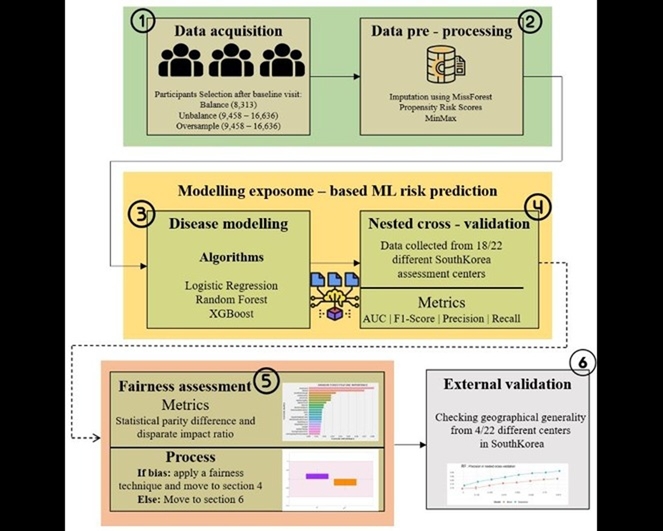

## 1 Introduction

The concept of the exposome, which encompasses lifelong environmental exposures and their effects on health [23], is gaining increasing attention in cardiovascular research. Montone et al. [12] discuss the exposome's relevance in ischemic heart disease, emphasizing its importance beyond traditional risk factors. Baragetti [1] further emphasizes the significance of the environmental exposome as a target for reimagining cardiovascular prevention, highlighting the need for comprehensive approaches. Daiber et al. [5] delve into the lifelong effects of environmental exposures on metabolism, health, and disease, providing insights into the long-term impact of the exposome. Hahad et al. [8] discuss how the environment can both pose problems and offer solutions, underscoring the complex interplay between environmental factors and health outcomes. Additionally, Hahad and Hahad y Al-Kindi [7] explore the prenatal and early life exposome's role in shaping health across the lifespan, highlighting the importance of early-life exposures. Stacy et al. [28] contribute by summarizing the evidence regarding the hazards induced by the exposome on children's health, providing a comprehensive overview of the plausibility database in this context. Together, these studies emphasize the multifaceted nature of the exposome and its profound implications for cardiovascular health and overall well-being across different life stages.

Cardiometabolic diseases, including cardiovascular disease (CVD), are increasingly recognized as being influenced by environmental factors. Schillemans et al. [19], Shahbazi y Byun [21] provide a comprehensive review of the role of per- and polyfluoroalkyl substances (PFAS) in cardiometabolic diseases, shedding light on the potential impact of these ubiquitous environmental contaminants on human health. Meanwhile, Sliwa et al. [25] focus on cardiovascular disease in low- and middle-income countries (LMICs) and its association with environmental factors. Their study underscores the global significance of environmental determinants in shaping cardiovascular health outcomes, particularly in regions where socioeconomic disparities and environmental pollution intersect [24], [10]. Together, these papers highlight the intricate interplay between environmental exposures and cardiometabolic health, emphasizing the need for further research and targeted interventions to address the environmental drivers of cardiovascular disease worldwide.

Recent research has delved into the intricate relationship between environmental exposures and cardiometabolic health, employing innovative approaches to predict disease risk and identify modifiable factors. Shahbazi et al. [22] presented a machine learning-based model that combines exposome data with electrocardiographic predictors to forecast diabetes risk, showcasing the potential of integrative approaches in disease prediction. Meanwhile, Poveda et al. [18] conducted an exposome wide ranking of modifiable risk factors for cardiometabolic disease traits, providing insights into the relative importance of various environmental exposures in disease development. Additionally, Lee et al. [11] employed questionnaire based exposome wide association studies (ExWAS) to uncover both expected and novel risk factors associated with cardiovascular outcomes, highlighting the utility of comprehensive exposome assessments in elucidating disease mechanisms and informing personalized prevention strategies. Together, these studies underscore the critical role of the exposome in shaping cardio-metabolic health and emphasize the need for interdisciplinary research efforts to address the complex interplay between environmental exposures and disease risk.

By acknowledging the different elements of the exposome, it becomes possible to identify specific pathways and gain valuable mechanistic knowledge regarding environmental exposures. This understanding enables us to grasp how everyday life, work, and commuting-related environmental exposures can impact the onset and advancement of cardiovascular disease (CVD). Furthermore, comprehending the interplay between the environment and cardiovascular health holds the potential to lay the groundwork for comprehensive preventive interventions on a large scale.

In the field of cardiovascular health, Münzel et al. [14] conducted a comprehensive expert review, examining various environmental risk factors and their association with cardiovascular diseases. This extensive review provides insights into the multifaceted nature of environmental influences on cardiovascular health, offering valuable knowledge for disease prevention and intervention strategies. Meanwhile, Vasyutina et al. [30] explored the utility of the zebrafish model system in dyslipidemia and atherosclerosis research, with a particular focus on environmental and exposome factors alongside genetic mechanisms. Their study demonstrates the relevance of integrating environmental and genetic approaches to unravel the complex etiology of cardiovascular diseases, showcasing the zebrafish model as a valuable tool for advancing our understanding of disease pathogenesis and identifying potential therapeutic targets. Together, these studies contribute to our understanding of the intricate interplay between environmental exposures, genetic predispositions, and cardiovascular health, emphasizing the importance of interdisciplinary research in addressing this critical public health issue.

Recent advancements in cardiovascular research have led to significant strides in precision prevention strategies aimed at reducing health disparities. Pearson et al. [16] discuss the science of precision prevention, highlighting research opportunities and clinical applications to address cardiovascular health disparities effectively. By integrating personalized approaches into prevention efforts, such as tailored interventions and risk assessment tools, this approach has the potential to mitigate disparities and improve outcomes for diverse populations. Complementing this, Holt et al. [9] focused on the development and validation of cardiovascular risk prediction equations in a large cohort of individuals with known cardiovascular disease. Their study underscores the importance of accurate risk prediction models in guiding preventive measures and treatment strategies, providing valuable insights into personalized cardiovascular care. Together, these studies underscore the transformative potential of precision prevention in cardiovascular medicine, offering hope for more equitable and effective approaches to reducing cardiovascular health disparities.

The exposome, encompassing various environmental and lifestyle factors, plays a significant role in shaping cardiovascular health outcomes. Münzel et al. [15] emphasize the substantial contribution of the exposome to the burden of cardiovascular disease, highlighting the need for comprehensive strategies to address environmental exposures in disease prevention. Building upon this, Motairek et al. [13] delve into the intricate relationship between the exposome and cardiovascular health, shedding light on the diverse array of exposures that impact cardiovascular outcomes. Their insights underscore the importance of understanding and mitigating environmental factors to promote cardiovascular well-being. Furthermore, Spatz et al. [27] advocate for prioritizing the exposome in efforts to reduce the burden of cardiovascular disease. By recognizing the exposome's role as a key determinant of cardiovascular health, they call for targeted interventions and policy initiatives aimed at mitigating environmental exposures and improving cardiovascular outcomes on a population level.

Most of the previous studies on the importance of identifying individuals at risk of cardiovascular disease (CVD) heavily relied on clinical data, which may not always be readily available or accessible to the general population. This limitation hindered their potential as effective self-assessment tools. Additionally, these studies overlooked the use of exposome data or only accounted for a restricted range of exposures. These studies utilized conventional linear modeling methods, including logistic regression, failed to fully harness the richness of exposome data, resulting in less accurate risk estimations.

To address these shortcomings, an innovative approach proposed for predicting CVD risk based on easily accessible exposome factors. Our research used extensive multi-center cohort data and underwent thorough internal and external validation through independent assessment centers. This study stands out as the first to investigate an extensive array of exposome data encompassing physical metrics, environmental factors, lifestyle influences, traumatic and psychosocial events, and early life conditions utilizing a substantial dataset.

Furthermore, the state-of-the-art employed for explainability, which allowed us to identify key exposome factors related to the occupation (present occupational status), completion of regular education enrollment, napping habits, dietary choices, and mental health (frequency of tiredness/tenseness). These factors were found to be significant risk indicators for CVD. This understanding could lead to the development of affordable preventive strategies and policies aimed at safeguarding individuals' health from detrimental environmental and life-style exposures [2]. Additionally, meticulously assessed the fairness of our proposed model concerning critical sensitive variables such as sex, ethnicity, and age, and found no evidence of bias. Moreover, performance evaluations conducted of the proposed model with an increasing number of features. Surprisingly, our model demonstrated comparable performance even when using as few as 40 exposome features for CVD risk prediction. This discovery significantly bolsters the capability of our model to serve as a swift and efficient risk assessment tool for self-evaluation.

The motivation for our study stands from the limitations in existing models that often overlook comprehensive exposome data, which can provide more accurate risk predictions. By identifying key exposome factors, we aim to bridge the gap between clinical data and environmental exposures, offering a more holistic view of CVD risk assessment. Our research addresses the critical need for inclusive and accessible tools for predicting cardiovascular disease, thereby empowering individuals to make informed health decisions.

In this study, we focus on enhancing the accuracy of cardiovascular risk predictions by incorporating a diverse range of exposome factors. Our motivation is driven by the need to integrate environmental data with traditional clinical metrics, providing a comprehensive framework for CVD risk assessment. Exposome data, encompassing lifestyle, environmental, and early-life exposures, is crucial in revealing the non-genetic factors that contribute to cardiovascular health. By including such data, our study highlights the dynamic interaction between an individual's environment and their biological predispositions, offering a more nuanced understanding of CVD risk. We aim to fill the gap in current research by offering insights into the multifaceted nature of cardiovascular health, emphasizing the importance of exposome data in understanding disease mechanisms.

Additionally, we are motivated by the growing public health demand for models that are not only scientifically rigorous but also accessible to a broader population. Many traditional CVD risk prediction tools require extensive clinical testing and biomarker analysis, which may not be available in low-resource settings. By using easily obtainable exposome factors, such as lifestyle habits and environmental exposures, we aim to develop a predictive model that can be applied in diverse healthcare contexts, especially where clinical resources are limited. This shift toward a more accessible and holistic risk prediction model has the potential to improve early interventions, allowing for targeted lifestyle modifications that can significantly reduce CVD risks.

Finally, we are driven by the opportunity to contribute to preventive health strategies. Cardiovascular disease is a leading cause of mortality worldwide, and identifying modifiable risk factors through exposome data offers a pathway to proactive, rather than reactive, healthcare. By focusing on environmental and lifestyle factors, our study aims to inform public health policies and individual behaviors, encouraging a preventative approach to managing cardiovascular risk. We believe that integrating these insights into clinical practice could lead to more personalized and effective interventions, ultimately reducing the global burden of cardiovascular diseases.

## 2 Materials and method

Prior to initiating model development, a careful selection process conducted to identify the most relevant exposome variables, grounded in their established relevance to cardiovascular disease and current data accessibility. This step was pivotal in ensuring the inclusion of a robust and comprehensive set of factors encompassing lifestyle, environmental, and biological domains. By adopting a meticulous approach to variable selection, the model's potential maximized to capture the multifaceted nature of CVD risk. Following this, the data was systematically structured to optimize integration within the machine learning framework, thereby ensuring high data quality and coherence. This preparatory phase not only enhanced the efficiency of subsequent modeling processes but also ensured that the model would focus on the most statistically and clinically significant variables, ultimately improving the accuracy and reliability of risk prediction.

**Figure 1:**
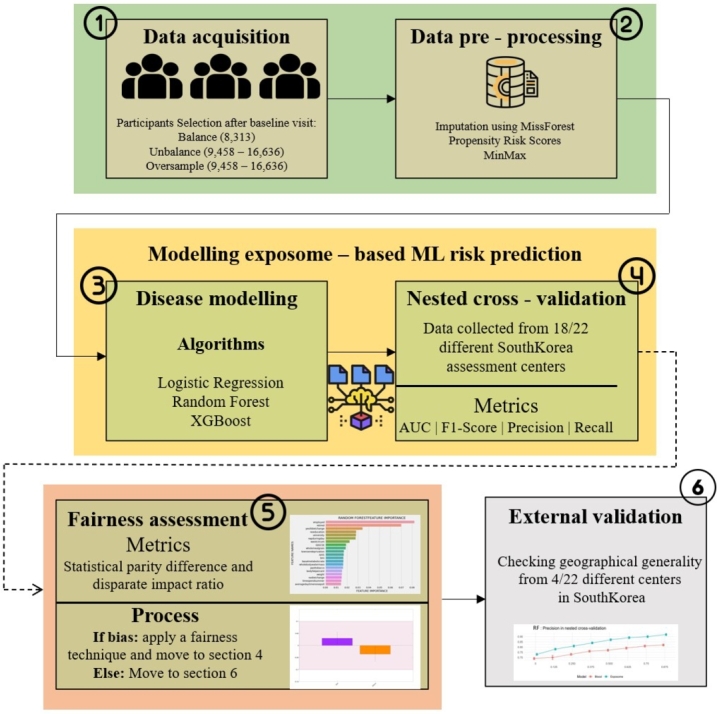
An outline of the suggested methodology for detecting individuals susceptible to CVD through the utilization of exposome data and machine learning.

Fig. 1 provides an overview of the proposed approach. Firstly sample population defined and selected the exposome variables to be included in the study. Next, data pre-processing conducted to handle missing data. The exposome-based ML model was then trained for CVD risk prediction. Validation of the model was carried out using both internal and external validation groups. Finally, the results analyzed and identified the most crucial exposome attributes contributing to disease prediction. Additionally, the model underwent evaluation for fairness to address potential biases. Each step in the pipeline is thoroughly explained in the subsequent sections.

### 2.1 Dataset

*Population.* The South Korean hospital records comprise a vast dataset collected from 250,100 participants who were recruited from the South Korea National Health Service between 2006 and 2019. The age range of the participants during recruitment was between 37 and 73 years. Over the course of the study, participants underwent up to four assessment visits, including a baseline visit conducted between 2006 and 2010, a first repeat visit from 2012 to 2013, an imaging visit from 2014 onwards, and a first repeat imaging visit from 2019 onwards. During the initial baseline visit, participants provided information on various aspects, including physical measurements, lifestyle choices, environmental factors, traumatic events, physiological events, early-life factors, and medical history. In subsequent visits, the medical history information was updated. To achieve the study's objective, exposome information collected during the first assessment visit from participants who did not have cardiovascular disease (CVD) at that time. Next, predicted the development of these respective pathologies based on subsequent visits' reported data. It is worth noting that these visits were conducted at 19 different clinical centers across South Korea. For external validation, data from three independent centers, different from those used for training and internal validation, were employed.

*Data pre-processing*

In the pre-processing stage, a thorough analysis was conducted on a total of 89 exposome features, categorized into six groups: early life (14 features), environmental factors (9 features), lifestyle (36 features), sociodemographics (10 features), mental health (11 features), and physical measures (9 features). Consequently, the final sample included 5,438 individuals with CVD after the baseline visit, matched by an equal number of participants without the studied diseases in the control group. Following the removal of certain participants, missing data were handled through imputation, with missing numerical values substituted by the median, while for categorical data, the most frequent values applied. To determine the appropriate imputation approach, sensitivity analysis conducted, which involved comparing models with imputed data using the MissForest algorithm, a popular and sophisticated imputation method, against simple imputation for different percentages of missing values (15%, 20%, 25%, 30%). Moreover, prior to imputation, variables with more than 30% missing data were excluded to ensure the robustness of the dataset and reduce bias introduced by excessive missingness. This threshold was determined through exploratory analysis, ensuring a balance between maintaining dataset integrity and maximizing the feature pool for model training. In this analysis, a paired t-test applied on the distributions of AUC (Area Under the Curve) performances within the nested cross-validation framework. The results indicated that there was no significant difference (p-value ≻ 0.05) between using missForest and simple imputation methods, thus leading us to adopt the latter approach.

### 2.2 Risk prediction based on exposome information

For identifying participants at risk of developing CVD, Random Forest (RF) employed, a robust machine learning algorithm known for its ability to achieve high predictive accuracy, especially in complex and high-dimensional problems involving heterogeneous data with both numerical and categorical features, as well as missing values. RF utilizes an ensemble learning approach by combining outputs from individual decision trees to reach a final decision. The machine learning (ML) model employed in this study for predicting cardiovascular disease (CVD) risk is based on a RF classifier, which is a well-established and widely used method in predictive modeling. the key components of the study, including the dataset used, validation methods, and performance metrics. Additionally, the study employs an advanced explainability method to elucidate the model's decision-making process, enhancing transparency. Further, feature importance analysis was conducted to identify the most influential variables in predicting CVD risk. This step was essential in refining the model, as it allowed for the exclusion of irrelevant or redundant factors, ultimately improving the efficiency and accuracy of the predictions. The resulting insights also contribute to a deeper understanding of which exposome factors play a crucial role in the onset of cardiovascular disease. The identification of significant exposome factors further contributes to the understanding of CVD risk assessment.

### 2.3 Internal and external validation

To evaluate our model's performance, both internal and external validations conducted. The external validation group consisted of participants randomly selected from three independent assessment centers, comprising 20% of the total study population. Additionally, we thoroughly examined whether the choice of centers influenced the model's performance and observed no significant variation when testing the model across different holdout centers. The internal validation group comprised participants from the other 19 centers. For this group, nested cross-validation was performed with seven outer folds. In each outer fold, the training set was further divided into five inner cross-validation folds to fine-tune the machine learning algorithm's hyperparameters. A grid search was employed to determine the optimal set of parameters and identify the best model for each outer fold. The chosen model was then evaluated using the external validation group.

In addition to grid search, feature selection was applied to reduce the dimensionality of the dataset, which further enhanced model performance. Principal Component Analysis (PCA) was employed to identify the most informative variables and remove redundant or noisy features. This step was crucial in refining the predictive model, allowing it to focus on the most critical exposome factors, ultimately leading to an increase in the overall accuracy and efficiency of the model.

Furthermore, the study also incorporated model fairness assessments, examining whether the model's predictions varied significantly across different demographic subgroups. Fairness metrics were calculated to ensure that the model did not disproportionately misclassify certain populations based on factors such as age, gender, or socioeconomic status. This analysis was pivotal in confirming that the model's application could be equitable across diverse populations, enhancing its potential for broad clinical implementation.

Next, adjusted various parameters of the RF model, including the learning rate, minimum child weight, gamma value, subsample, subsampling of columns per tree, and maximum depth. These parameters were selected from a range of predefined values. Specifically, the learning rate was chosen from [0.05, 0.10, 0.15, 0.20, 0.25, 0.30], the minimum child weight from [1, 5, 10], gamma value from [0.5, 1, 5], subsample from [0.6, 0.8, 1.0], subsampling of columns per tree from [0.6, 0.8, 1.0], and maximum depth from [3, 4, 5]. The RF model was implemented using Python 3.8 with the Scikit-Learn library version 1.0.

To assess the proposed model's performance and compare it with reference models, sensitivity, specificity, precision, and the area under the receiver operating characteristic curve (AUC) utilized as evaluation metrics.

### 2.4 Interpretability of the proposed model

A significant aspect of this study's contribution is the identification of exposome attributes that may be modifiable and play a crucial role in predicting the risk of CVD. These attributes hold the potential to be targeted for lifestyle and exposure interventions, with a focus on preventing cardiovascular disease. To achieve this objective, feature extraction values utilized [20], [4] to extract and determine the most important features. This method employs cooperative game theory to estimate the contribution of each feature towards the prediction process.

Moreover, understanding the interactions between various exposome factors can enhance the predictive power of cardiovascular disease models. By integrating data from different sources, such as environmental monitoring and individual health records, researchers can gain a more comprehensive view of how these factors interact and contribute to CVD risk. This holistic approach not only improves prediction accuracy but also allows for the identification of high-risk individuals who may benefit most from targeted interventions.

## 3 Results

The results of this study reveal significant associations between modifiable exposome attributes and the risk of cardiovascular disease (CVD). Through comprehensive feature extraction and analysis, several key factors emerged as critical predictors of CVD risk, highlighting the importance of lifestyle and environmental interventions. Notably, the data demonstrated that certain behavioral patterns, along with exposure to specific environmental elements, significantly influenced cardiovascular health outcomes. These findings underscore the potential for targeted strategies aimed at reducing exposure and promoting healthier lifestyle choices to mitigate CVD risk.

### 3.1 Existing models comparison

The risk models incorporated 89 exposome factors, deliberately excluding medical data, biomarkers (e.g., blood tests), and other variables that are not commonly accessible in daily life, such as diastolic and systolic blood pressure, impedance, arm fat mass, and treatment or medication details. Notably, 54 exposome factors used were not part of the most comprehensive biological and clinical model proposed by Pomares-Millan [17], including those related to environmental, early-life, and mental health factors. The focus was to estimate disease risk using only readily accessible exposome data, aiming for a more personalized and interpretable approach. Many of these factors are modifiable, offering opportunities for intervention. Furthermore, the algorithm's performance in predicting cardiovascular disease (CVD) risk was compared to the well-established Framingham risk score [12], which relies on traditional risk factors such as age, sex, LDL cholesterol, HDL cholesterol, systolic blood pressure, diabetes, and smoking. These same factors were used to train a random forest (RF) machine learning model to predict CVD risk, following the same experimental setup as the exposome-based model.

Two types of dataset used in this process which one of them is exposome data and the other is clinical data. The patient is the same person who provided the exposome and clinical data to hospital. To analyze these datasets and to achieve the good results we have used three different ways as mentioned balanced, unbalanced and oversample which the results of each of them separately presented in tables and figures.

### 3.2 CVD risk individuals

During each iteration, the machine learning model was trained using 2,752 subjects from each class, comprising both the control group and individuals at risk of cardiovascular disease (CVD). Afterward, 855 subjects were employed for internal validation, while 408 subjects were utilized for external validation.

Table 1 shows a comparison between sensitivity, specificity, precision, and AUC (Area Under the Curve) to evaluate the performance of various models based on internal validation. These models include (i) the proposed machine learning exposome-based model, (ii) the clinical-based model, and (iii) the traditional Framingham risk score.

**Table 1:** Comparison results between balanced, unbalanced and over-sample approaches (internal validation).

Model	AUC	F1-Score	Precision	Recall
AI model: Random Forest (Balanced)				

Exposome	0.72 (+/-) 0.01	0.72 (+/-) 0.02	0.72 (+/-) 0.01	0.71 (+/-) 0.02
Blood	0.68 (+/-) 0.01	0.67 (+/-) 0.01	0.69 (+/-) 0.01	0.64 (+/-) 0.02
Framingham score	0.66 (+/-) 0.01	0.80 (+/-) 0.01	0.69 (+/-) 0.01	0.43 (+/-) 0.01

AI model: Random Forest (Unbalanced)				

Exposome	0.72 (+/-) 0.01	0.67 (+/-) 0.02	0.72 (+/-) 0.01	0.64 (+/-) 0.03
Blood	0.67 (+/-) 0.01	0.61 (+/-) 0.01	0.68 (+/-) 0.03	0.55 (+/-) 0.02
Framingham score	0.63 (+/-) 0.01	0.59 (+/-) 0.01	0.65 (+/-) 0.01	0.51 (+/-) 0.01

**AI model: Random Forest (Oversample)**				

Exposome	**0.86 (+/-) 0.01**	**0.85 (+/-) 0.01**	**0.93 (+/-) 0.01**	**0.78 (+/-) 0.01**
Blood	**0.83 (+/-) 0.01**	**0.83 (+/-) 0.01**	**0.84 (+/-) 0.02**	**0.82 (+/-) 0.01**
Framingham score	**0.80 (+/-) 0.01**	**0.80 (+/-) 0.01**	**0.79 (+/-) 0.01**	**0.77 (+/-) 0.01**

Using the proposed exposome-based model, an AUC of 86—83% (internal—external) for accurately identifying individuals at risk of cardiovascular disease (CVD) during the internal—external validation achieved. This performance represents a statistically significant improvement (p-value = 0.01) over the traditional Framingham score, which demonstrated limited performance in this specific cohort (AUC of 66—64%).

**Figure 2:**
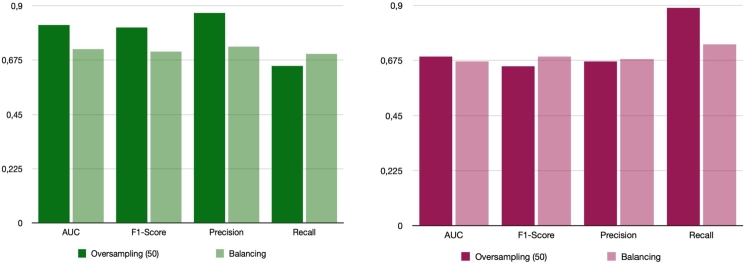
Receiver Operating Characteristic Area Under the Curve (AUC, F1-Score, Precision, and Recall) values for predicting cardiovascular disease (CVD) risk using the proposed exposome-based machine learning (ML) model are provided for the internal (left) and external (right) validation groups for balanced and oversampled dataset.

Fig. 2 shows the comparison of the balancing and oversampling exposome-based and clinical-based approaches outperform the ML model based on Random Forest variables. Furthermore, the proposed model, which solely relies on exposome data, demonstrated a behavior comparable to the Alaa et al. model, and there was no statistically significant difference in their performance in terms of AUC (p-value ≻ 0.05). Subsequently, a more comprehensive model was developed using all available features, including exposome and clinical information. This model showcased superior performance compared to all other models, achieving an AUC of 88% and 86% for internal and external validation, respectively. However, it is important to note that this comprehensive model cannot be used for self-assessment. The highest weight in CVD prediction comes from prescribed medications, which serve as an actual indicator of the diseases. Consequently, these medications are the primary contributors to the model's impressive performance.

**Figure 3:**
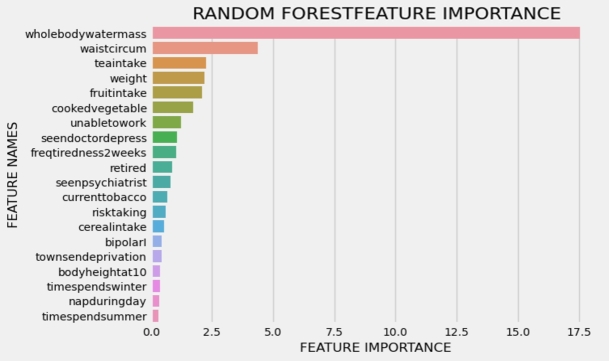
Interpreting the exposome-based model (balanced data), presents the top 20 essential factors in predicting the risk of cardiovascular disease (CVD), ranked by their mean absolute value.

**Figure 4:**
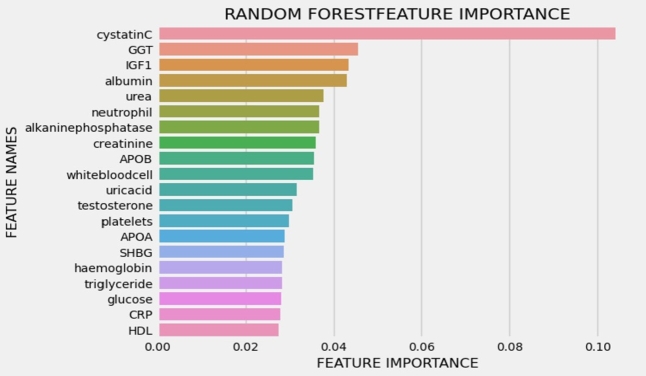
Interpreting the clinical data (balanced data), the panel presents the top 20 essential factors in predicting the risk of cardiovascular disease (CVD), ranked by their mean absolute value.

Figure 3, Figure 4 illustrate the 20 key variables crucial in predicting the risk of cardiovascular disease (CVD) using the exposome-based model and clinical data on balanced data. The CVD prediction is significantly influenced by various sociodemographic factors, including employment status, material deprivation measured by the Townsend index, level of qualifications, daytime napping, and the extent of completed full-time education. Lifestyle choices, such as dietary patterns, sleep duration, coffee preference (decaffeinated, instant, ground), and tobacco use, also exhibit associations with CVD risk. Remarkably, findings from previous research [29] align with these results. Notably, aspects related to mental well-being, like the frequency of tiredness and tenseness, are among the most significant contributors in identifying individuals at risk of developing CVD.

Table 2 presents the performance analysis of balanced data over ML algorithms with 8318 sample size.

**Table 2:** Performance analysis of the balancing data with 8318 sample size (internal validation).

Model	AUC	F1-Score	Precision	Recall
AI model: XGBoost				

Exposome	0.72 (+/-) 0.01	0.71 (+/-) 0.01	0.73 (+/-) 0.01	0.70 (+/-) 0.02
Blood	0.68 (+/-) 0.01	0.67 (+/-) 0.01	0.69 (+/-) 0.01	0.69 (+/-) 0.02
Exposome + Blood	0.70 (+/-) 0.01	0.69 (+/-) 0.01	0.73 (+/-) 0.01	0.69 (+/-) 0.02

AI model: Logistic Regression				

Exposome	0.72 (+/-) 0.01	0.72 (+/-) 0.02	0.72 (+/-) 0.01	0.71 (+/-) 0.02
Blood	0.68 (+/-) 0.01	0.67 (+/-) 0.01	0.69 (+/-) 0.01	0.64 (+/-) 0.02
Exposome + Blood	0.71 (+/-) 0.01	0.69 (+/-) 0.01	0.70 (+/-) 0.01	0.70 (+/-) 0.02

**AI model: Random Forest**				

Exposome	**0.73 (+/-) 0.01**	**0.72 (+/-) 0.02**	**0.74 (+/-) 0.01**	**0.71 (+/-) 0.02**
Blood	0.67 (+/-) 0.01	0.65 (+/-) 0.01	0.69 (+/-) 0.01	0.62 (+/-) 0.01
Exposome + Blood	**0.73 (+/-) 0.01**	**0.71 (+/-) 0.01**	**0.75 (+/-) 0.01**	**0.71 (+/-) 0.02**

**Figure 5:**
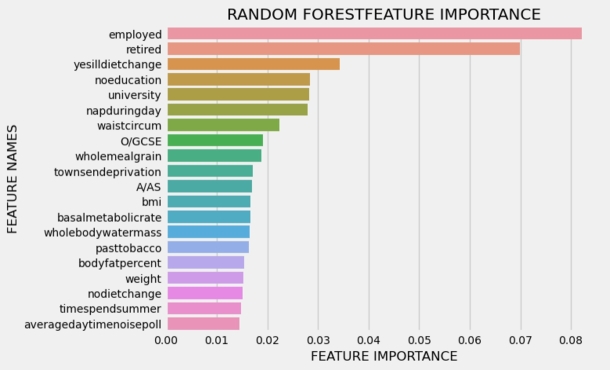
Participants baseline characteristics used for disease prediction (internal—external validations of oversample group (exposome)). The number of individuals and their mean and standard deviation are reported.

**Figure 6:**
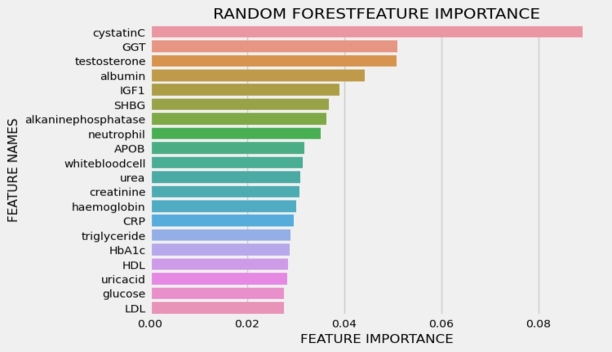
Participants baseline characteristics used for disease prediction (internal—external validations of oversample group (clinical)). The number of individuals and their mean and standard deviation are reported.

Figure 5, Figure 6 illustrate the 20 key variables crucial in predicting the risk of cardiovascular disease (CVD) using the exposome-based model and clinical data on oversample data. Table 3 presents the performance analysis of oversample data over ML algorithms with a 12478 sample size, and Table 4 presents the performance analysis of unbalanced data with a 10398 sample size.

**Table 3:** Performance analysis of the oversampling data with 12478 sample size (internal validation).

Model	AUC	F1-Score	Precision	Recall
AI model: XGBoost				

Exposome	0.82 (+/-) 0.01	0.81 (+/-) 0.01	0.87 (+/-) 0.01	0.75 (+/-) 0.01
Blood	0.76 (+/-) 0.01	0.75 (+/-) 0.01	0.77 (+/-) 0.01	0.73 (+/-) 0.01
Exposome + Blood	0.81 (+/-) 0.01	0.80 (+/-) 0.01	0.85 (+/-) 0.01	0.75 (+/-) 0.02

**AI model: Random Forest**				

Exposome	**0.82 (+/-) 0.01**	**0.81 (+/-) 0.01**	**0.88 (+/-) 0.01**	**0.75 (+/-) 0.01**
Blood	**0.79 (+/-) 0.01**	**0.78 (+/-) 0.01**	**0.80 (+/-) 0.02**	**0.79 (+/-) 0.01**
Exposome + Blood	**0.82 (+/-) 0.01**	**0.82 (+/-) 0.01**	**0.87 (+/-) 0.01**	**0.76 (+/-) 0.02**

AI model: Logistic Regression				

Exposome	0.74 (+/-) 0.01	0.74 (+/-) 0.01	0.75 (+/-) 0.01	0.73 (+/-) 0.02
Blood	0.68 (+/-) 0.01	0.67 (+/-) 0.01	0.70 (+/-) 0.01	0.64 (+/-) 0.02
Exposome + Blood	0.73 (+/-) 0.01	0.73 (+/-) 0.01	0.72 (+/-) 0.01	0.72 (+/-) 0.02

**Table 4:** Performance analysis of the unbalancing data with 10398 sample size.

Model	AUC	F1-Score	Precision	Recall
AI model: XGBoost				

Exposome	0.69 (+/-) 0.01	0.58 (+/-) 0.02	0.69 (+/-) 0.01	0.50 (+/-) 0.03
Blood	0.65 (+/-) 0.01	0.50 (+/-) 0.01	0.66 (+/-) 0.02	0.40 (+/-) 0.01
Exposome + Blood	0.67 (+/-) 0.01	0.57 (+/-) 0.01	0.67 (+/-) 0.01	0.48 (+/-) 0.02

**AI model: Random Forest**				

Exposome	**0.70 (+/-) 0.01**	**0.60 (+/-) 0.02**	**0.65 (+/-) 0.02**	**0.55 (+/-) 0.02**
Blood	0.65 (+/-) 0.01	0.51 (+/-) 0.01	0.63 (+/-) 0.02	0.43 (+/-) 0.01
Exposome + Blood	**0.69 (+/-) 0.01**	**0.58 (+/-) 0.01**	**0.64 (+/-) 0.01**	**0.54 (+/-) 0.02**

AI model: Logistic Regression				

Exposome	0.69 (+/-) 0.01	0.58 (+/-) 0.02	0.69 (+/-) 0.02	0.50 (+/-) 0.02
Blood	0.64 (+/-) 0.01	0.48 (+/-) 0.01	0.68 (+/-) 0.02	0.37 (+/-) 0.01
Exposome + Blood	0.69 (+/-) 0.01	0.57 (+/-) 0.01	0.68 (+/-) 0.01	0.49 (+/-) 0.02

**Figure 7:**
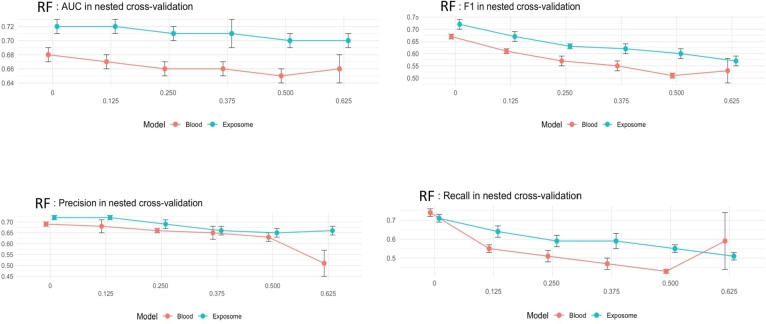
Model performance based on the exposome and clinical data using nested cross-validation for CVD risk prediction over unbalanced group.

**Figure 8:**
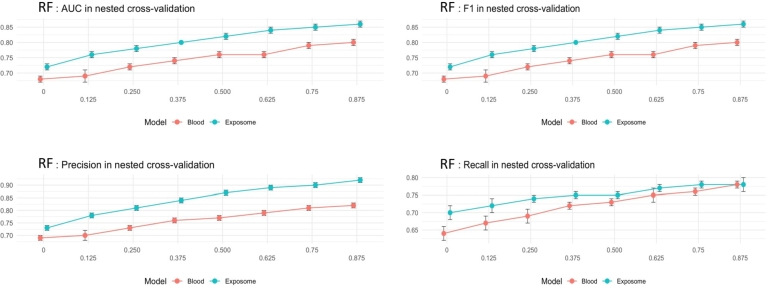
Model performance based on the exposome and clinical data using nested cross-validation for CVD risk prediction considering an increasing number of features (oversampled group).

Figure 7, Figure 8 present the results of unbalanced and oversampled approached using nested cross-validation over AUC, F1-score, precision, and recall.

**Figure 9:**
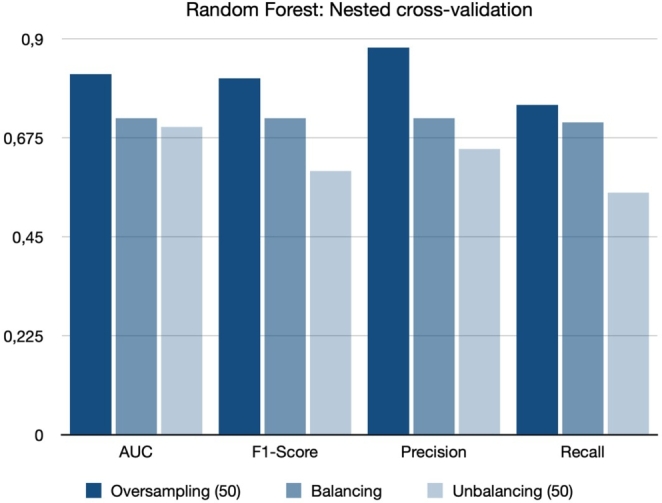
Operating characteristic area under the curve (AUC, F1-Score, Precision, and Recall) values for predicting cardiovascular disease (CVD) risk using the proposed exposome-based machine learning (ML) model are provided for the balancing, unbalancing, and oversampling groups.

Fig. 9 presents the comparison of the nested cross-validation for the balanced, unbalanced, and oversampled data. In this Figure, the oversampled case clearly contains better results compared with balanced and unbalanced. The balanced data take the second stage and the unbalanced data has the lowest results in all the cases of AUC, F1-score, precision, and recall.

### 3.3 A streamlined exposome-based model utilizing a concise selection of features

The evaluation of the exposome-based models conducted for predicting CVD. This evaluation involved using an increasing number of the most significant features, as indicated by the Gini importance score of the model Fig. 8. The findings demonstrated that even with only the 40 or 20 most crucial features, our model achieved comparable performance (measured by AUC, precision, and sensitivity) to using the entire set of exposome features for CVD prediction.

**Figure 10:**
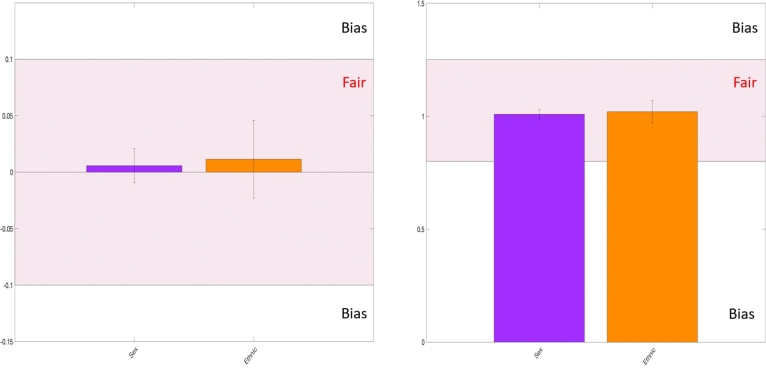
Fairness performance of the exposome-based model for CVD prediction of the balancing group. Models are deemed fair when their statistical parity difference falls within the range of -0.1 to 0.1, indicating a balanced distribution across different groups. Similarly, fair models exhibit disparate ratios between 0.75 and 1.25, showing equitable treatment across sensitive attributes. It's essential to highlight that this evaluation specifically pertains to individuals aged between 20 and 50 years.

**Figure 11:**
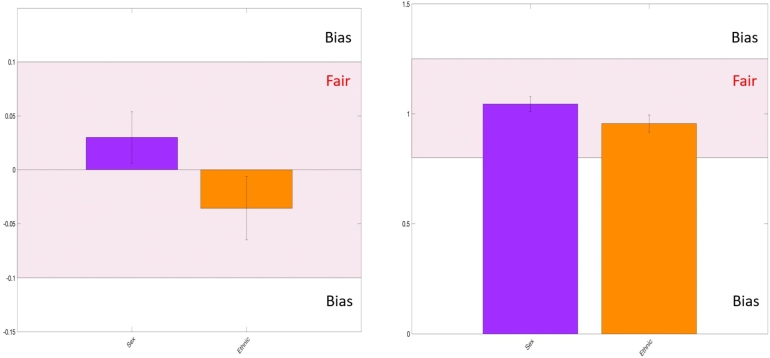
Fairness performance of the exposome-based model for CVD prediction of the unbalancing group. Models are deemed fair when their statistical parity difference falls within the range of -0.1 to 0.1, indicating a balanced distribution across different groups. Similarly, fair models exhibit disparate ratios between 0.75 and 1.25, showing equitable treatment across sensitive attributes. It's essential to highlight that this evaluation specifically pertains to individuals aged between 20 and 50 years.

Furthermore, the algorithmic fairness of the proposed models examined for CVD risk prediction. To assess fairness, the statistical parity difference computed [6] and the disparate impact ratio [6]. The results are presented in Figure 10, Figure 11. Notably, no bias was identified concerning any of the sensitive variables considered, such as ethnicity, gender, or age. It is important to mention that assessed age-related bias specified by evaluating two age groups: one comprising individuals aged 20 to 50 years and another corresponding group of older individuals.

## 4 Policy and practical implications

This study's findings have significant policy implications for public health and cardiovascular disease prevention. By demonstrating the importance of incorporating exposome data into CVD risk prediction models, the study highlights the need for policymakers to consider environmental and lifestyle factors as crucial determinants of cardiovascular health. Policies aimed at reducing environmental pollution, promoting healthy lifestyle choices, and improving early-life conditions could play a vital role in mitigating cardiovascular disease risk on a population level. Moreover, the study supports for integrating exposome data into national health guidelines and preventive care strategies, encouraging healthcare systems to extract more comprehensive approach to cardiovascular risk assessment that goes beyond traditional clinical metrics. Policymakers are motivated to prioritize investments in exposome research and data collection, enabling more effective public health interventions that address the root causes of cardiovascular disease. From a practical point, the study offers valuable insights into the development of user-friendly self-assessment tools for individuals to evaluate their cardiovascular risk. By leveraging exposome data, these tools can provide personalized risk profiles, empowering individuals to make informed lifestyle and health decisions. Healthcare practitioners can also benefit from this approach by gaining access to a more detailed understanding of their patients' risk factors, leading to more targeted and effective preventive measures. Furthermore, the study's emphasis on exposome data can inform workplace wellness programs and community health initiatives, encouraging organizations to consider environmental factors in their efforts to promote cardiovascular health.

## 5 Discussion

Through the utilization of a sizable population cohort in South Korea, two innovative machine learning risk prediction models that rely on exposome-based data created and verified. These models are designed to identify individuals who may be susceptible to a significant disease, namely cardiovascular disease (CVD). Our approach involves incorporating various exposome features from different categories, thereby eliminating the need for costly, laborious, and time-consuming clinical information acquisition. The utilization of easily accessible exposome features underscores the potential of our model to serve as a rapid assessment tool, even facilitating self-assessment. Only a limited amount of research has utilized exposome factors to predict the risk of cardiovascular disease (CVD). Notably, Montone et al. [12] demonstrated high accuracy in their work by incorporating biological and clinical markers, along with a few exposure predictors. However, they did not consider early-life factors. In contrast, our proposed approach achieved similar performance to their integrative model, despite relying solely on readily available exposome factors, including early-life factors. The external validation resulted in area under the curve (AUC) values of 0.82 (+/-) 0.01 for CVD. The exposome-based model proposed for predicting cardiovascular disease (CVD) risk demonstrated superior performance compared to the widely-used Framingham risk score. The key features that had a significant impact include: (i) sociodemographic factors, such as employment or education status (e.g., current job status, age at completion of full-time education), (ii) physical measurements, including waist circumference, (iii) mental health factors, like the frequency of fatigue and tension, and (iv) lifestyle habits, such as smoking, daytime napping, alcohol intake, and dietary patterns. Interestingly, these findings align with risk factors that have been clinically reported in the existing literature [26]. Furthermore, the robustness of the exposome-based model proved with just a few selected features, as illustrated in Fig. 9. As a result, simpler versions of the CVD model can implemented by using around 40 or 20 features, making it more user-friendly and reducing the amount of information users need to provide, without significantly compromising its performance. Although our findings are of significant importance, there are limitations in our current work. The South Korea hospital records cohort had a considerable number of missing values for some exposome variables, a common challenge in longitudinal studies. To address this limitation, a model capable empolyed for handling missing data and adopted a straightforward yet effective imputation approach for both categorical and numerical data [3].

Moreover, it is essential to acknowledge that our study was predominantly based on a white population from South Korea. To assess the general applicability and transferability of our proposed risk prediction model, further research is warranted using cohorts from diverse countries and ethnic backgrounds. This will allow us to evaluate the broader relevance of our findings.

## 6 Conclusion

The influence of the exposome on genetic effects is substantial, accounting for approximately 70 to 90% of the risk associated with major diseases. Consequently, developing new approaches to predict the risk of these illnesses based on the human exposome presents a promising opportunity for advancing early prevention strategies and encouraging beneficial lifestyle modifications. An innovative study was undertaken to explore the potential of the human exposome in identifying individuals at risk for cardiovascular disease (CVD), a major health condition. The study utilized a comprehensive set of exposome data, encompassing sociodemographic, lifestyle, environmental, occupational, psychosocial, mental, and early-life factors. By integrating machine learning techniques with a large-scale population cohort, the research demonstrated the power of an exposome-based model as a valuable tool for personalized, accessible risk prediction in future healthcare. Given these findings, policymakers should prioritize initiatives that integrate exposome-based insights into public health strategies. This includes creating policies that encourage the reduction of harmful environmental exposures and promote healthier living conditions. Governments and healthcare providers should invest in community programs that educate individuals about the exposome's impact on health, while also implementing regulations to limit exposure to known environmental hazards. Furthermore, it is crucial to support research and innovation in exposome science to continually refine predictive models and ensure they are accessible to diverse populations. By encouraging collaboration across sectors, policymakers can develop comprehensive solutions that address both individual and societal health risks, ultimately leading to more effective prevention and intervention strategies for cardiovascular diseases.

## 7 Future research direction

The study opens several paths for future research in the field of cardiovascular health and exposome studies. Future investigations could explore the longitudinal effects of exposome variables on cardiovascular outcomes, assessing how congested environmental exposures impact disease progression over time. Additionally, research could focus on specific subpopulations, such as individuals with genetic predispositions to CVD, to evaluate the interplay between genetic and environmental factors. The development of more advanced machine learning models that incorporate real-time exposome data, such as wearable sensors and IoT devices, represents another promising direction for future research. Furthermore, cross-disciplinary studies involving epidemiology, environmental science, and data analytics could enhance our understanding of the exposome's role in cardiovascular health, leading to more effective prevention and intervention strategies. Finally, research efforts should aim to refine and validate the proposed models in diverse populations, ensuring their applicability and accuracy across different demographic and geographic contexts.

## CRediT authorship contribution statement

**Zeinab Shahbazi:** Writing – review & editing, Writing – original draft, Validation, Methodology, Formal analysis, Data curation. **Slawomir Nowaczyk:** Supervision, Funding acquisition.

## Declaration of Competing Interest

The authors declare that they have no known competing financial interests or personal relationships that could have appeared to influence the work reported in this paper.

## Data Availability

Not applicable.
